# Supplementary figures and images for: Influence of Cane Molasses Inclusion to Dairy Cow Diets during the Transition Period on Rumen Epithelial Development
Source: Animals (Basel). 2021 Apr 24;11(5):1230. doi: 10.3390/ani11051230 (PMC8145355; doi:10.3390/ani11051230)

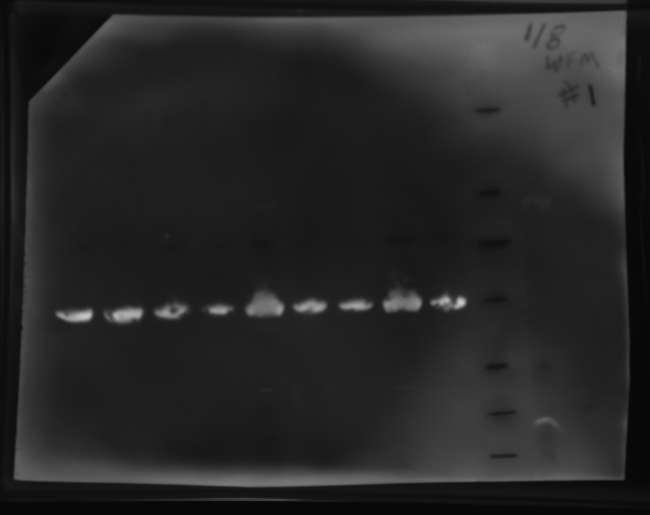

Supplement: Supplementary file 1 [file animals-11-01230-s001.zip › Figure S1.tif]

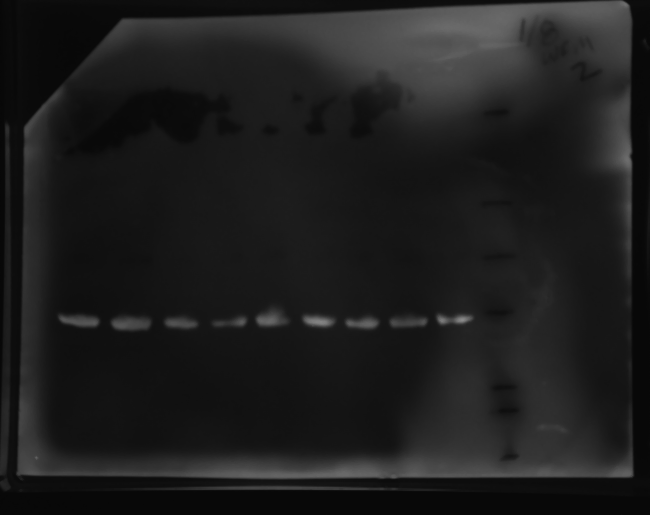

Supplement: Supplementary file 1 [file animals-11-01230-s001.zip › Figure S2.tif]
